# Lattice Strain Relaxation and Compositional Control in As-Rich GaAsP/(100)GaAs Heterostructures Grown by MOVPE

**DOI:** 10.3390/ma16124254

**Published:** 2023-06-08

**Authors:** Paola Prete, Daniele Calabriso, Emiliano Burresi, Leander Tapfer, Nico Lovergine

**Affiliations:** 1 Institute of Microelectronics and Microsystems of CNR (IMM-CNR), Lecce Unit, Via Monteroni, I-73100 Lecce, Italy; 2 Department of Innovation Engineering, University of Salento, Via Monteroni, I-73100 Lecce, Italy; danielecalabriso.le@gmail.com; 3 ENEA—National Agency for New Technologies, Energy and Sustainable Economic Development, Brindisi Research Center, Strada Statale 7 ‘Appia’, I-72100 Brindisi, Italy; emiliano.burresi@enea.it (E.B.); ltapfer@yahoo.com (L.T.)

**Keywords:** GaAsP, III-V heterostructures, strain relaxation, critical thickness, metastable heterostructures, metalorganic vapor phase epitaxy, solid-vapor segregation coefficient, high-resolution X-ray diffraction, field emission scanning electron microscopy, III-V based solar cells

## Abstract

The fabrication of high-efficiency GaAsP-based solar cells on GaAs wafers requires addressing structural issues arising from the materials lattice mismatch. We report on tensile strain relaxation and composition control of MOVPE-grown As-rich GaAs_1−x_P_x_/(100)GaAs heterostructures studied by double-crystal X-ray diffraction and field emission scanning electron microscopy. Thin (80–150 nm) GaAs_1−x_P_x_ epilayers appear partially relaxed (within 1−12% of the initial misfit) through a network of misfit dislocations along the sample [011] and $\left[01\overset{-}{1}\right]$ in plane directions. Values of the residual lattice strain as a function of epilayer thickness were compared with predictions from the equilibrium (Matthews–Blakeslee) and energy balance models. It is shown that the epilayers relax at a slower rate than expected based on the equilibrium model, an effect ascribed to the existence of an energy barrier to the nucleation of new dislocations. The study of GaAs_1−x_P_x_ composition as a function of the V-group precursors ratio in the vapor during growth allowed for the determination of the As/P anion segregation coefficient. The latter agrees with values reported in the literature for P-rich alloys grown using the same precursor combination. P-incorporation into nearly pseudomorphic heterostructures turns out to be kinetically activated, with an activation energy E_A_ = 1.41 ± 0.04 eV over the entire alloy compositional range.

## 1. Introduction

Multi-junction (tandem) semiconductor solar cells in the form of stacked single-junction cells, each absorbing a different interval of the solar spectrum, allow for external quantum efficiencies beyond the Shockley–Queisser limit for single-junction cells [1,2,3]. Tandem solar cells based on a crystalline silicon (Si) bottom junction are very attractive due to the relative low cost of Si. A dual-junction cell with a 1.7 eV top junction based on GaAsP and a Si (1.12 eV) bottom cell raises the theoretical power conversion efficiency (PCE) of the tandem cell above 30%. A common approach to the fabrication of such tandem cells is the direct monolithic growth of the III-V cell onto the Si bottom cell. However, structural constraints between III-V compounds and Si (e.g., the combined effects of lattice, thermal, and crystal polarity mismatches) limit the performances of such cells; despite the tremendous improvements in III-V/Si heteroepitaxy over recent years [4,5], solar PCEs remain far from theoretical figures [6,7].

Alternative approaches are being studied to overcome these limitations. Among others, the combination of a top cell based on free-standing III-V nanowires with a planar Si bottom cell could guarantee higher efficiencies than their monolithic planar counterparts due to their potentially higher structural quality, as the lattice mismatch in these nanostructured cells can be accommodated more easily than in planar structures. In addition, nanowires have great potential as super-absorptive media for the fabrication of efficient solar cells [8]. Self-assembly of III-V nanowires on Si has been demonstrated [9] as well as tandem cells [10,11], while multiband absorption has been proposed using intermediate-band dilute-nitride III-V nanowires [12]. However, nanowire self-assembly is a complex process [13,14], and nanowire-based solar cells are still in an early stage of development.

Four-terminal tandem cells composed of a thin III-V planar cell mechanically stacked onto an interdigitated back contact Si cell with a glass interlayer have recently shown efficiency above 32.6% [15,16]. The advantage of such an approach is that the III-V top-cell could be monolithically grown on GaAs with higher quality: indeed, the 1.7 eV direct-bandgap GaAs_0.775_P_0.225_ (As-rich) alloy has +8.18 × 10^−3^ lattice misfit with GaAs, against the −2.28 × 10^−2^ of Si; similarly, the estimated RT thermal mismatch between GaAsP and GaAs is <3.8%, against values in the 110−118% range for GaAsP/Si heterostructures [17,18]. In addition, as-grown III-V structures are not affected by antiphase domains and rotational twins as in the heteroepitaxy on Si [4,19]. Chemical lift-off of the cell from the underlying GaAs and multiple re-utilization of the substrate have been demonstrated [20] as viable strategies to keep production costs low [21]. Wafer-bonded triple-junction III–V//Si solar cells with a PCE of 35.9% were also demonstrated by this approach [22].

Despite the abovementioned advantages of fabricating the GaAsP-based top-cell on GaAs, a compositionally graded GaAsP buffer is usually adopted between the substrate and the cell heterostructure to limit defect generation and propagation (e.g., threading dislocations, TDs) within the device. Indeed, GaAsP- and InGaAs-based metamorphic solar cells have been demonstrated on suitably step-graded GaAsP/(100)GaAs structures [23]. Low (10^4^–10^6^ cm^−2^) densities of TDs have been reported in GaAsP/(100)GaAs heterostructures grown by metalorganic vapor phase (MOVPE) [24] or molecular beam epitaxy [25]. Still, studies on the actual relaxation rate with thickness of single GaAs_1−x_P_x_ (*x* < 0.5) epilayers on GaAs have never been published before in the literature. 

This work reports for the first time on the lattice strain and plastic relaxation (misfit dislocation formation) of MOVPE-grown GaAs_1−x_P_x_/(100)GaAs heterostructures investigated by high-resolution double-crystal X-ray diffraction and field emission scanning electron microscopy (FESEM). The relaxation rate of present samples is estimated and compared with theoretical predictions; observations are explained in terms of dislocation nucleation. Furthermore, the As/P anion segregation coefficient for the GaAs_1−x_P_x_ alloy is determined for our growth conditions and found to be in agreement with previous estimates at lower growth temperatures. The work will help to better understand the epitaxy of metamorphic step-graded GaAsP/(100)GaAs heterostructures as virtual substrates for the growth of high-efficiency GaAsP-based top solar cells.

## 2. Materials and Methods

GaAs_1−x_P_x_ thin epilayers were grown on device-quality vertical gradient freeze (100)GaAs substrates (Wafer Technology, Milton Keynes, UK) by low (50 mbar) pressure MOVPE using an Aix 200RD reactor (Aixtron, Herzogenrath, Germany). Trimethyl-gallium (Me_3_Ga), tertiary-butyl-arsine (^t^BuAsH_2_), and tertiary-butyl-phosphine (^t^BuPH_2_) (Dockweiler Chemicals, Marburg, Germany) were employed as Ga, As, and P precursors, respectively. Before loading into the reactor, the substrates were cleaned in isopropanol vapors for 1 h, etched in a H_2_SO_4_:H_2_O_2_:H_2_O (4:1:2) solution for 8 min at around 40 °C, thoroughly rinsed in de-ionized water, and finally dried under pure N_2_. In-situ annealing of the substrates was then performed for 10 min at 625 °C under a H_2_ + ^t^BuAsH_2_ atmosphere to desorb oxides, organic residuals, and other contaminants from the GaAs surface. A thin (7 nm) GaAs epilayer was grown at the same temperature to reconstruct the substrate surface before GaAsP growth. Upon completion of the GaAs epilayer growth, the reactor temperature was lowered to 600 °C in a H_2_ + ^t^BuAsH_2_ flow. GaAsP epilayers were grown under a fixed Me_3_Ga molar flow of 12.3 μmol/min to ensure the same growth rate (~0.085 nm/s) for all samples. Different concentrations of V-group elements in the vapor were adopted to study the effects on GaAsP composition: the vapor stoichiometry *x*_v_ = [^t^BuPH_2_]/([^t^BuPH_2_] + [^t^BuAsH_2_]) was varied between 0.46 and 0.60, while the V:III ratio was fixed at 20:1 or 40:1. The growth time was in the 15–30 min range, so to obtain GaAsP thickness around one hundred nm. 

The sample surface morphology was investigated by field-emission scanning electron microscopy (FESEM) in-plan observations using a Sigma VP (Zeiss, Oberkochen, Germany) microscope equipped with a Gemini-1 electron column and a primary electron beam energy of 20 keV. To this end, secondary (SE) or backscattered electron (BSE) signals were employed.

The microstructural properties of GaAs_1−x_P_x_ alloy epilayers were investigated by X-ray diffraction. The measurements were carried out using an Empyrean diffractometer (Malvern-Panalytical, Malvern, UK) in a high-resolution double-crystal (HRDC) configuration. A Cu-target was employed as an X-ray source, and a 4-bounce Ge-crystal Bartels monochromator-collimator (symmetrical (220)-reflections) with an angular divergence of about 14 arcsec was employed as X-ray incidence optics. All measurements were carried out using a “wide open” PIXcel detector. The strain state and alloy composition of GaAs_1−x_P_x_ epilayers were determined by recording symmetrical and asymmetrical HRDC measurements in the vicinity of the (400) and (422) lattice points, respectively. In order to account for a possible offcut of the substrate surface or a tilt/rotation of the epilayer with respect to the substrate, all measurements were performed at four azimuthal angle settings (i.e., sample rotations about the surface normal), namely φ = 0, π/2, π, and 3/2π, corresponding to the X-ray scattering plane along the in-plane 〈110〉 directions. For the asymmetric (422) reflection, the geometrical configuration with high incidence and glancing exit angles, i.e., direction cosines $\gamma_{0}>\left|\gamma_{h}\right|$, was chosen because this configuration is very sensitive to in-plane strain components and lattice relaxation.

## 3. Results and Discussion

### 3.1. Determination of Lattice Strain and Alloy Composition of GaAs_1−x_P_x_ Epilayers from HRDC

**Figure 1:**
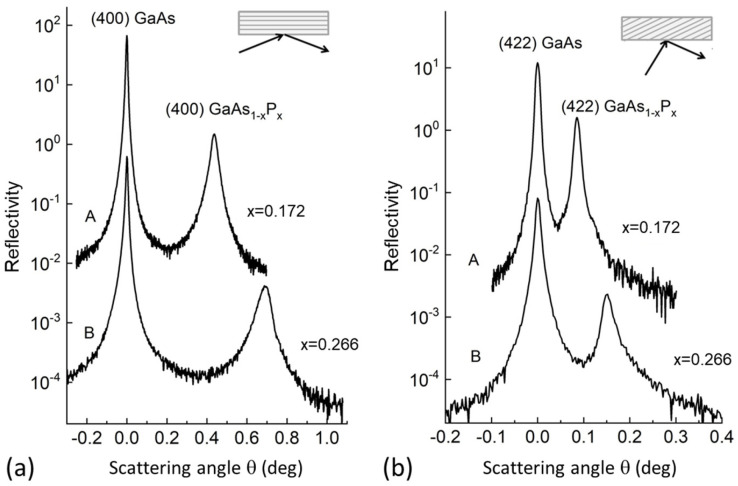
(**a**) Symmetrical (400) HRDC patterns recorded for two GaAs_1−x_P_x_/(100)GaAs heterostructures having GaAs_1−x_P_x_ alloy composition x = 0.172 (sample A) and x = 0.266 (sample B). (**b**) Asymmetrical (422) HRDC patterns in the glancing exit setting, $\gamma_{0}>\left|\gamma_{h}\right|$ (see inset), recorded for the same samples in (**a**). A schematic of the diffraction geometry is reported in the upper-left part of each panel.

Figure 1 shows the HRDC patterns around the symmetrical (400) and asymmetrical (422) reflections of two GaAs_1−x_P_x_/(100)GaAs heterostructures having different phosphorous mole fractions *x*. Patterns measured for the different azimuthal angles (not reported here) demonstrate that all epilayers are nearly pseudomorphic. Measured values of GaAs_1−x_P_x_ lattice strain parallel to the (100) interface plane ($\epsilon_{∥}$) are summarized in Table 1 for the studied samples. The lattice mismatch (*f*) and mole fraction *x* in the Table were calculated by using the relations of the second-order approximation for 〈100〉-oriented zinc-blende heterostructures [26]. For the calculations, the lattice parameters and elastic constants of GaAs and GaP reported by Adachi [27] were used. The samples show different amounts of strain $\epsilon_{∥}$ and thus different degrees of plastic relaxation $\delta_{∥}\equiv \left(f-\epsilon_{∥}\right)/f$ within the 1−12% range.

### 3.2. Observation of Defects-Related Features in GaAs_1−x_P_x_ Epilayers through FESEM

**Figure 2:**
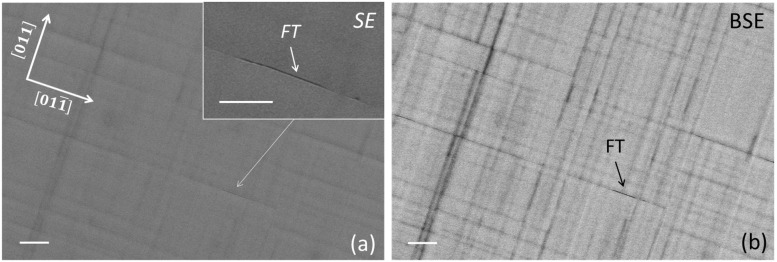
Plan-view FESEM micrographs of Sample C recorded by using the microscope (**a**) SE and (**b**) BSE current signals. A short-faceted trench (FT) indicated by the arrow is observed in (**a**) and better visualized in the magnified micrograph shown in the inset. The same FT is observed as a few-micron long and narrow black segment in (**b**). White markers in the micrographs represent 4 μm.

Figure 2a shows a FESEM surface micrograph of Sample C ($\delta_{∥}$ = 11.3%) obtained using SE imaging (sensitive to surface morphology); it shows the presence of mutually perpendicular (i.e., along the sample [011] and $\left[01\overset{-}{1}\right]$ in-plane directions) undulations of the epilayer surface, so-called cross-hatch morphology, observed in low-misfit GaAsP layers grown at relatively low temperatures [28]. The presence of cross-hatch is explained as the combination of strain relaxation by dislocation nucleation at surface steps (and their subsequent glide into the epilayer) and growth by surface step flow, which tends to smooth down the steps [29]. FESEM observations of the same surface region using BSE imaging show dense patterns of well-resolved mutually perpendicular dark lines (Figure 2b), corresponding (both in position and alignment) to the surface undulations in Figure 2a. As the samples are compositionally homogeneous and BSE imaging is less sensitive to surface features, these lines originate most likely from electron channeling contrast imaging (ECCI) associated with crystal defects (e.g., dislocations) [30,31,32]. Besides the surface cross-hatch, we observed the seldom occurrence of faceted trenches (FTs) (inset of Figure 2a) aligned along the $\left[01\overset{-}{1}\right]$ direction with lengths varying between a few microns and several hundred microns. The latter have been associated with the formation of a micro-twin at the FT cusp [25]; interestingly, a strong BSE imaging contrast is observed in Figure 2b at a FT location. In comparison, BSE micrographs of Sample A (not reported here) show a negligible (although not null) density of dark lines, in agreement with the reduced plastic relaxation of this sample ($\delta_{∥}$ = 1.78%).

### 3.3. Analysis of Epilayer Strain Relaxation

**Figure 3:**
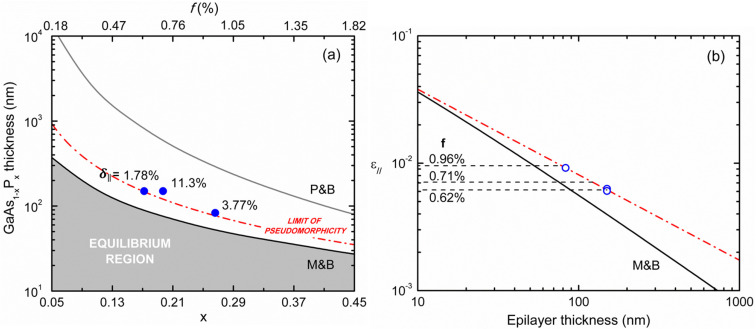
(**a**) GaAs_1−x_P_x_ thickness as function of alloy composition for the analyzed samples (blue points) and values of the critical thickness for plastic relaxation, calculated according to Matthews-Blakeslee (M&B) [33] and People-Bean (P&B) [34] (Appendix A). The percentage of plastic relaxation ($\delta_{∥}$) is also indicated for each sample in the diagram. The dash-dotted red curve represents values of $h_{c}^{eff}$ (see main text). (**b**) Lattice strain $\epsilon_{∥}$ as function of epilayer thickness for the measured samples (light blue points). Solid black line: expected values of $\epsilon_{∥}(h)$ according to M&B (Equation (A2)). The dashed horizontal lines represent the sample misfit values (Table 1). Dash-dotted red line in (**b**): Equation (1) with parameter values *A* = 0.178 ± 0.038 and *m* = 0.671 ± 0.046, best-fitting the experimental points.

Figure 3a reports the GaAs_1−x_P_x_ thickness (Table 1) along with values of the critical thickness ($h_{c}$) for strain relaxation calculated based on the equilibrium theory of Matthews-Blakeslee [33] (Appendix A) as a function of alloy compositions. It appears that the epilayer thickness is beyond the corresponding $h_{c}$ value, in qualitative agreement with the sample partial relaxation observed by HRDC. However, more compelling information on strain relaxation behavior in present heterostructures can be obtained by comparing the calculated Matthews–Blakeslee residual strain $\epsilon_{∥}$ for ${h>h}_{c}$ (Equation (A2)) with that measured in our samples as a function of the epilayer thickness, as shown in Figure 3b. The diagram clearly shows that the present epilayers are less plastically relaxed (metastable) than expected based on the equilibrium theory. This is a common experimental finding in mismatched heterostructures grown on high-crystalline-quality substrates (VGF-grown GaAs in our case), i.e., whenever the substrate TD density is not large enough to generate the required amount of plastic relaxation; new misfit dislocations must be then nucleated during the growth, a process limited by energy balance or kinetic barriers. The first case was proposed by People and Bean [34], who estimated the energy threshold for the generation of screw dislocations in a strained epilayer (Appendix A), despite the fact that such dislocations cannot relax elastic strain. Figure 3a reports the critical thickness for strain relaxation as a function of GaAs_1−x_P_x_ composition based on the People–Bean model (Equation (A3)): the as-estimated values of $h_{c}$ appear indeed much larger than those calculated from Matthews–Blakeslee theory and well beyond the thickness of our partially relaxed epilayers. Plastic relaxation has been described by Marée et al. [35] in terms of surface nucleation and expansion into the epilayer of dissociated half-loops, taking into account the work done by the elastic stress field acting on expanding loops. This model was found to agree fairly well with experimental strain relaxation data in compressively-strained heterostructures [36].

For sufficiently thick epilayers, the observed dependence of the residual strain with thickness can be fitted by the semi-empirical power-law function

(1) $$\epsilon_{∥}\left(h\right)=Ah^{-m},$$

where *m* = 1 for the Matthews–Blakeslee theory and *m* = 1/2 for the energy balance models [34,35] (Appendix A). The latter value is in good agreement with relaxation data for compressively strained metastable heterostructures [36,37]. Best-fitting of experimental data in Figure 3b with Equation (1) returned instead, a value *m* = 0.671 ± 0.046 (i.e., *m*~ 2/3). This finding suggests a relaxation rate behavior intermediate between that of Matthews–Blakeslee and the half-loop nucleation models; indeed, a larger proclivity toward plastic relaxation is expected for tensile-strained epilayers with respect to compressive ones.

Finally, we estimate the apparent critical thickness ($h_{c}^{eff}$) for strain relaxation of GaAsP/(100)GaAs heterostructures as a function of alloy composition by imposing the pseudomorphicity condition $\epsilon_{∥}=f$ to the quantity $\epsilon_{∥}\left(h\right)$ (Equation (1)) best fitting our experimental data. Figure 3a shows that the Matthews–Blakeslee $h_{c}$ values lie below the $h_{c}^{eff}$ curve, while all experimental points lie above it. Clearly, the $h_{c}^{eff}$ curve represents an upper bound to epilayer pseudomorphicity in reason of the limited resolution (1 × 10^−4^) of HRDC strain measurements; indeed, the absence of measurable strain does not imply that misfit dislocations are not present, as they would be generated as soon as the energy conditions allow it, that is, well before strain relaxation becomes appreciable. In this sense, electron microscopy observations (e.g., through ECCI) of individual dislocations are necessary to verify whether the onset of relaxation coincides with that of Matthews–Blakeslee or if it occurs at a larger thickness.

To date, studies on the structural properties of step-graded GaAs_1−x_P_x_ buffer layers for solar cell applications have predominantly focused on the evaluation of TD and FT densities as functions of the step compositional height and grading rate (i.e., the compositional change per unit thickness of the grown alloy): a lower grading rate was shown necessary with increasing x to maintain the density of FTs low and reduce the TD density [24,25]. However, no particular attention was paid to the actual strain relaxation within each of the buffer grading steps in those studies, despite the fact that the actual degree of plastic relaxation would affect the distribution of TDs throughout the final buffer layer. The present findings will help in further optimizing the structural properties of such step-graded GaAsP buffer layers, as well as in properly engineering strain-balanced InGaAs/GaAsP multiple quantum well structures as current-matched light-absorbing medium in monolithic triple-junction InGaAs/GaAs/Ge solar cells [38,39], ultimately leading to better performance III-V tandem solar cells. 

Tensile-strained GaAsP layers also find applications in the fabrication of InGaAsP quantum well-based laser diode heterostructures on GaAs for NIR photon emission [40,41]. In this case, the use of an InGaAsP/InGaAsP/GaAsP active region allows for an effective reduction of non-radiative recombination within the device and suppression of carrier leakage with respect to conventional AlGaAs/GaAs heterostructure laser diodes [42]. As the mechanism of degradation in a laser diode is related to the development of dark line defects associated with the generation and multiplication of misfit dislocations within the heterostructure active region, understanding GaAsP relaxation behavior is therefore critical in ensuring suppression/reduction of plastic relaxation within the proposed laser device structures.

### 3.4. Determination of the Solid-Vapor Segregation Coefficient for GaAs_1−x_P_x_

**Figure 4:**
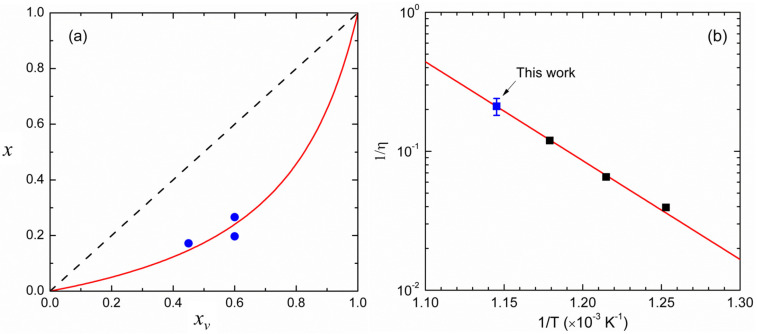
(**a**) Solid-vapor distribution diagram for the MOVPE growth of the GaAsP alloy at 600 °C: solid blue points represent experimental data; the red curve is the solid-vapor distribution curve (Equation (2)) best-fitting the experimental points with η = 4.76 ± 0.66; (**b**) Arrhenius plot of 1/η as a function of the growth temperature: data are from the best-fitting value in (**a**) (solid blue point) and values reported in ref. [44] (solid black points).

Figure 4a reports the solid-vapor distribution diagram for the analyzed GaAsP epilayers. It can be clearly observed that the P-composition *x* in the solid alloy is always below the corresponding content in the vapor (*x*_v_) during the sample growth. The relative distribution of As and P between the two phases is described by the so-called segregation coefficient *η* defined as [43]

$$\eta =\frac{N_{As}/N_{P}}{\left[tBuAsH_{2}\right]/\left[tBuPH_{2}\right]},$$

where $N_{As}/N_{P}$ represents the As to P anion concentration ratio in the GaAsP alloy and $\left[tBuAsH_{2}\right]/\left[tBuPH_{2}\right]$ is the corresponding precursor concentration ratio in the vapor. *η* has been shown to depend on the nature of the employed precursors and the growth temperature [43,44]. Furthermore, preferential As (P) segregation was observed for tensile (compressive) strained GaAsP epilayers with respect to fully relaxed ones, a compositional latching phenomenon ascribed to the different radii of As and P anions [28,45]. 

As $x\equiv N_{P}/\left(N_{P}+N_{As}\right)$, its value can be calculated for a given *x*_v_ composition of the vapor by the following expression:

(2) $$x=1/\left[\eta \left(\frac{1}{x_{v}}-1\right)+1\right],$$

if the actual value of *η* is known. We best-fitted the experimental points in Figure 4a with Equation (2) in order to determine *η* for our experimental conditions, which turned out to be 4.76 ± 0.66. As *η* > 1, a preferential As incorporation in the GaAsP alloy occurs indeed in our nearly pseudomorphic (tensile-strained) samples. Figure 4b allows us to compare our best-fitting *η* value with those estimated at lower growth temperatures by Chen et al. [44] for the same V-group precursor combination: the Arrhenius plot shows that the 1/*η* values align almost perfectly (regression coefficient R = 0.9985), indicating that P incorporation into the crystal is kinetically activated (i.e., increases with the growth temperature), with an apparent activation energy E_A_ = 1.41 ± 0.04 eV, not far from that (1.23 ± 0.05 eV) estimated in ref. [44]. Noteworthy is also that very thin (20−40 nm) GaAs_1−x_P_x_ (0.91 < *x* < 1.0) epilayers were employed by those authors for their estimation, indicating that the observed temperature dependence of η in Figure 4b holds across the entire compositional range.

## 4. Conclusions

We reported on tensile strain relaxation and composition control of MOVPE-grown GaAs_1−x_P_x_/(100)GaAs heterostructures studied by HRDC X-ray diffraction measurements and FESEM observations. The strain values and alloy P-compositions were measured by HRDC, while FESEM observations proved the presence of misfit dislocations and their effect on the epilayer surface morphology. Thin (80–150 nm) GaAs_1−x_P_x_ epilayers appear partially relaxed through a network of misfit dislocations along the sample [011] and $\left[01\overset{-}{1}\right]$ in-plane directions, giving rise to a cross-hatch surface morphology. 

The relaxation rate as a function of epilayer thickness was compared with theoretical predictions from equilibrium (Matthews–Blakeslee) and energy balance models. It was shown that present epilayers relax at a slower rate than predicted by the equilibrium model, an effect ascribed to the existence of an energy barrier to the nucleation of new dislocations. A relaxation rate behavior intermediate between that of Matthews–Blakeslee and the half-loop nucleation models is proposed, although further data over a larger compositional interval are needed to confirm this finding. 

The analysis of As-rich GaAs_1−x_P_x_ alloy composition as a function of V-group precursors ratio and growth temperature allowed to determine the As/P anion segregation coefficient and compare it with previous reports in the literature. P incorporation into the crystal turned out kinetically activated, with an apparent activation energy E_A_ = 1.41 ± 0.04 eV over the entire alloy compositional range.

The present results will help to optimize the design and growth of metamorphic GaAsP/(100)GaAs heterostructures as virtual substrates for the epitaxy of high-efficiency GaAsP-based solar cells and InGaAsP/InGaAsP/GaAsP-based NIR-emitting laser diodes.

## Figures and Tables

**Table 1:** Composition (*x*) and elastic strain parallel to the hetero-interface ($\epsilon_{∥}$) measured through HRDC for the investigated GaAs_1−x_P_x_/(100)GaAs heterostructures. Values of the epilayer thickness (*h*) and calculated lattice misfit (*f*) are also reported for each sample.

Sample	GaAs_1−x_P_x_ Thickness, *h* (nm)	*x*	Misfit, * *f* (×10^−3^)	$\mathrm{Lattice}\;\mathrm{Strain},\;*\;\epsilon_{∥}\;(\times 10^{-3})$
A	150	0.172	6.17	6.06
B	83	0.266	9.55	9.19
C	150	0.197	7.10	6.30

* Defined as $f=\left(a_{GaAs}-a\right)/a$ and $\epsilon_{∥}=\left(a_{∥}-a\right)/a$, where $a_{GaAs}$ is the GaAs bulk lattice parameter, whilst *a* and $a_{∥}$ are the GaAs_1−x_P_x_ bulk and strained lattice parameters in the direction parallel to the heteroepitaxial interface, respectively.

## Data Availability

The data presented in this study are available on request.

## Appendix A

Plastic relaxation in strained heterostructures occurs through the generation of a network of dislocation lines (so-called misfit dislocations) lying on the epilayer/substrate interface plane; each misfit dislocation is supposed to be either (i) generated from the stretching and bending of pre-existing (i.e., in the substrate) TDs by the epilayer elastic stress field or (ii) nucleated anew during the epilayer growth. The two mechanisms have been alternatively employed in heterostructure relaxation models based on energy minimization or energy balance considerations, respectively.

According to Matthews–Blakeslee equilibrium theory [33], the critical thickness ($h_{c}$) for the onset of plastic relaxation in mismatched heterostructures can be calculated upon minimization of the epilayer total (elastic and plastic) areal energy density, assuming the misfit dislocations are generated by mechanism (i) above, resulting in the equation

(A1) $$h_{c}=\frac{b}{4\pi f\cos\lambda }\left(\frac{1-\nu \;\cos^{2}\lambda }{1-\nu }\right)\left[1+ln\left(h_{c}/b\right)\right],$$

where ν is the Poisson ratio of the epilayer crystal (for GaAsP alloys, ν ≈ 0.31 [46,47]), *b* is the Burger vector of the misfit dislocations, and λ is the angle between the Burger vector and the normal to the dislocation line. For thickness values $h<h_{c}$ the minimal energy is obtained for fully pseudomorphic epilayers ($\epsilon_{∥}=f$), while for $h>h_{c}$ plastic relaxation is possible and the elastic strain shall decrease according to the expression

(A2) $$\epsilon_{∥}(h)=\frac{b}{4\pi h\cos\lambda }\left(\frac{1-\nu \;\cos^{2}\lambda }{1-\nu }\right)\left[1+ln\left(h/b\right)\right].$$

It appears from Equation (A2) that for large thickness (h/b≫1) one obtains $\epsilon_{∥}(h)\sim h^{-1}$.

In (100)-oriented semiconductor heterostructures, plastic relaxation occurs mostly through a network of 60°-mixed perfect dislocations along [011] and $\left[01\overset{-}{1}\right]$ in-plane directions, having Burger vectors $\vec{b}=\frac{a}{2}\langle 011\rangle$ (*a* being the epilayer lattice parameter), their edge-component parallel to the epilayer/substrate interface being $b_{∥}=b\cos\lambda$, and λ = 60°. Given the heterostructure misfit *f*, the critical thickness $h_{c}$ and elastic strain $\epsilon_{∥}(h)$ can be readily calculated from Equations (A1) and (A2), respectively.

Alternative relaxation models have been proposed to estimate the critical thickness in mismatched heterostructures based on energy balance considerations [34,35]. In the People–Bean model, misfit dislocations are generated when the areal elastic energy density of the strained epilayers exceeds the energy density required for the generation of a screw dislocation at the epilayer/substrate hetero-interface; the critical thickness $h_{c}$ can be then estimated by solving the equation [34].

(A3) $$h_{c}=\frac{1}{16\pi \sqrt{2}a(x)}{\left(\frac{b}{f}\right)}^{2}\left(\frac{1-\nu }{1+\nu }\right)ln\left(h_{c}/b\right),$$

where *a*(*x*) is the epilayer lattice parameter, and the other symbols have the same meaning as above. For $h>h_{c}$ plastic relaxation is expected, and the elastic strain decreases according to the expression

$$\epsilon_{∥}(h)=\frac{b}{4\pi \sqrt[{4}]{2}}\sqrt{\frac{\pi }{a(x)}\left(\frac{1}{h}\right)\left(\frac{1-\nu }{1+\nu }\right)ln\left(h/b\right)},$$

For large thickness values, this again leads to $\epsilon_{∥}(h)\sim h^{-1/2}$. The same functional dependence is obtained by the half-loop dislocation nucleation model [35].
